# Pruritus after continuous administration of epidural morphine for post-cesarean delivery analgesia: a case control study

**DOI:** 10.1186/s12884-020-03522-6

**Published:** 2021-01-15

**Authors:** Xinyi Tian, Kaifan Niu, Hong Cao, Gonghao Zhan, Yan Zhang, Feng Xu, Wangning Shangguan, Ye Gao

**Affiliations:** 1 Department of Anesthesia, Pain and Critical Care Medicine, The Second Affiliated Hospital and Yuying Children’s Hospital of Wenzhou Medical University, Wenzhou, 325027 China; 2 West China School of Medicine, West China Hospital, Sichuan University, Chengdu, 610041 P. R. China; 3 School of Public Health, Fudan University, Shanghai, China; 4 NHC Key Lab. of Reproduction Regulation (Shanghai Institute of Planned Parenthood Research), Fudan University, Shanghai, China

**Keywords:** Pruritus, Risk factors, Continuous epidural morphine, Pain, Ropivacaine, Levobupivacaine

## Abstract

**Background:**

Pruritus is one of the most common side effects of epidural morphine administered for post-surgery analgesia, and pregnant women tend to be highly susceptible. The relative contributions of morphine concentration, local anesthetics, and level of pain to pruritus after epidural morphine for post-cesarean delivery analgesia remain unclear. Accordingly, the present study aimed to identify risk factors for pruritus after continuous administration of epidural morphine for post-cesarean delivery analgesia.

**Methods:**

This case control study was based on routinely collected clinical data. Participants included women who had undergone cesarean section and adopted a patient-controlled analgesia pump for postoperative analgesia. A series of logistic regression analyses were performed. Interaction terms were added to explore the moderation effects of combined local anesthetics and pain level on associations between morphine concentration and pruritus. Robustness of the results was checked through sensitivity analysis using propensity scores matching approach.

**Results:**

Higher morphine concentration, assisted reproductive treatment, and multipara and cesarean section history were significantly more prevalent in the pruritus group than in the control group. The probabilities of pruritus at morphine concentrations of 10, 15, 20, 25, 30 and 40 μg/mL increased sequentially from 0.05, 0.1, 0.2, 0.35, 0.54 to 0.84, respectively. The trend remained steep in the ropivacaine stratum and became flatter when combined with levobupivacaine. At mild pain combined with levobupivacaine, the incidence of pruritus increased from 0.33 (95% confidence interval [CI] 0.1–0.68) in the 10 μg/mL morphine group to 0.48 (95% CI 0.1–0.88) in the 40 μg/mL morphine group. In the stratum of moderate pain combined with levobupivacaine, the incidence increased from 0.4 (95% CI 0.04–0.92) to 0.56 (95% CI 0.03–0.98). The results in the sensitivity analysis were in consistent with above findings.

**Conclusions:**

Higher concentrations of morphine, multipara, and assisted reproductive treatment were factors associated with a higher probability of pruritus. Pain level or combined local anesthetics could moderate the association between morphine concentration and pruritus.

## Background

Epidural morphine can provide effective and long-lasting analgesia due to its rapid onset and sustained effect of pain relief, and is commonly used as an analgesic after cesarean section [1, 2]. However, the adverse effects of opioid administration, such as epidural morphine, including urinary retention, reduced mobility [3], pruritus, nausea and vomiting [4, 5], are often inevitable. It is important to achieve a satisfactory balance between pain reduction and acceptable side effects in clinical practice.

Pruritus is described as a subjective unpleasant and irritating sensation that often promotes uncontrollable scratching [5]. This feeling of discomfort is a common side effect of neuraxial opioids [6], and parturients appear to be the most susceptible group. It occurs in up to 60% of patients after epidural morphine for delivery analgesia, and the incidence may be even higher after subarachnoid injection of morphine [7–9]. Although pruritus is not a life-threatening complication [9], it is highly disturbing and may further lead to skin ulceration, sleep disturbance, anxiety or depression, even increase length of hospital stay, psychological-related accidents and injuries, especially when pruritus is severe [10–13]. As such, pruritus warrants significant clinical attention [14].

Morphine dosage in clinical applications is widely considered to affect the incidence or severity of pruritus, although the results of existing studies are not consistent. Studies by Palmer in 1999 [15] and Nermin in 2008 [16] revealed that, as the dose of morphine increased, so did the incidence of pruritus. Another study reported that as the dose of morphine increased, the severity of pruritus increased [17]. However, there are also studies that indicate pruritus is not related to morphine dosage. Jiang et al. found that, although pruritus in the morphine group was more common than in the control group, there was no significant association between the incidence of pruritus and morphine dosages [18]. In addition, opioids in combination with local anesthetics are currently considered to be an ideal mixture for epidural analgesia in labor [8, 19]. It is generally believed that pruritus is a side effect of the combined use of neuraxial morphine and local anesthetics as analgesia. However, little is currently known about whether combined local anesthetics have an impact on the incidence of pruritus.

The purpose of this study was to identify risk factors for pruritus after continuous administration of epidural morphine for post-cesarean delivery analgesia. The primary research question was whether the occurrence of pruritus after cesarean analgesia is related to the amount of morphine use. The secondary research question was whether the combination of local anesthetics or the level of pain affects the association between pruritus and morphine.

## Methods

### Study design, site, and population

The present investigation was a retrospective, observational, case-control study based on routinely collected clinical data. Participants were selected from a group of patients who had undergone cesarean section and adopted a patient-controlled analgesia pump for postoperative analgesia at the Second Affiliated Hospital and Yuying Children’s Hospital of Wenzhou Medical University (Wenzhou, Zhejiang, China) between June 2011 and March 2014. Given the retrospective nature of the study and the use of anonymized patient data, requirements for informed consent were waived and it received approval from the ethical committee. The women enrolled had undergone non-emergent cesarean section with continuous epidural anesthesia or combined spinal-epidural anesthesia and adopted a patient-controlled analgesia pump for postoperative analgesia. Women who underwent general anesthesia or intravenous analgesia pump (non-epidural), analgesic pump without morphine, had missing information regarding analgesia pump use, those who underwent emergent cesarean section, with coexisting preoperative pruritic diseases, previous allergy history, and American Society of Anesthesiologist (ASA) class III or higher than ASA anesthesia classification III were excluded.

### Implementation and data collection

Women enrolled in this study received either epidural or spinal-epidural anesthesia before cesarean section. If epidural anesthesia was administered, the anesthesiologist would set an epidural catheter and inject 1% lidocaine plus 0.375% ropivacaine or levobupivacaine through the catheter until the level of anesthesia was at T4. If spinal epidural anesthesia was administered, the anesthesiologist would inject 0.5% ropivacaine into the subarachnoid space and leave a catheter in the epidural space to strengthen analgesia during or after surgery. Epidural analgesia pump was set to a volume of 100 mL and contained 0.15% ropivacaine or 0.15% levobupivacaine and different concentrations of morphine. An initial loading dose consisted of 1 mg morphine plus 8–10 mL of same solution of the pump. The automatic dosing rate was 2 mL/h and the patient-controlled dosing was 2 mL with a lock-out dosing interval of 15 min. Postoperative follow-up was completed by anesthesiologists in the Acute Pain Group 24 h after the surgery. Follow-up information was recorded in the electronic medical record system. Data analyzed in this study, which included age, maternal and infant health information, ASA grade, information regarding analgesia pump use, operation time, operation type and records of complaints regarding pruritus, were independently extracted by two trained researchers.

### Measurements

#### Pruritus

The primary outcome measure of this study was pruritus, which was defined an uncomfortable itching that occurred within 24 h after cesarean section, and required intervention from health care providers (with records in electronic medical systems). Pruritus was recorded as a binary variable (i.e., 0 = no reported pruritus; 1= reported pruritus).

#### Morphine concentration

Morphine concentration refers to the ratio of morphine dosage (1–4 mg) to the volume of analgesic pump (100 mL), and was recorded as a continuous variable (μg/mL).

#### Combination of local anesthetics

The analgesic pump contained either ropivacaine or levobupivacaine, and was recorded as a binary variable (1 = ropivacaine; 2 = levobupivacaine).

#### Pain level

Pain level was defined as the most severe pain within 24 h after cesarean section and scored by using 11-point numerical rating scale. It was recorded as a four-level ordinal categorical variable: none (= 0), mild (> 0 but ≤ 3), moderate (> 3 but ≤ 6) and severe (> 6 but ≤ 10).

Other variables included in the analysis included maternal age (0, < 35 years; 1, ≥ 35 years). Body mass index: (low, < 18.5 kg/m^2^; normal, 18.5–24.9 kg/m^2^; overweight, 25–29.9 kg/m^2^; and obese, ≥ 30 kg/m^2^); gestational diabetes (0 = without gestational diabetes; 1 = with gestational diabetes); hypertension during pregnancy: (0 = without pregnancy hypertension; 1 = with pregnancy hypertension); primipara (0 = multipara; 1 = primipara); previous cesarean section (0 = no history of cesarean section; 1 = history of cesarean section); and assisted reproductive treatment, which referred to whether the woman received in vitro fertilization (IVF) for the current pregnancy (0 = no; 1 = yes).

### Sample size

Sample size in the present unmatched case-control study was calculated with a two-sided 95% confidence level (i.e., 1 – alpha), as follows: power (% chance of detection) 80%; ratio of controls to cases 2:1; hypothetical proportion of cases with exposure 20%; and hypothetical proportion difference 5%. The calculated sample size was 73. Given the actual sample size of 201 versus 402, the power was estimated to be > 0.80 using the method described by Fleiss, Tytun, and Ury [20].

### Analytical approach

#### Statistical analysis

Continuous data with symmetric distributions are expressed as mean and standard deviation (SD). Continuous data with skewed distributions are expressed as median and the 25th–75th percentile (i.e., interquartile range [IQR]). Categorical data are reported as count and percentage (%). Differences between the study and control groups were analyzed using the Kruskal-Wallis rank sum test for continuous nonparametric data and the χ^2^ test for categorical data. The Spearman correlation matrix was used to identify highly correlated variables due to possible multicollinearity. Differences with *P* < 0.05 (*P* < 0.10 for interaction terms) were considered to be statistically significant.

#### Association analysis

The first step was to conduct a series of univariate binary logistic regression analyses. At the level of *P* < 0.10, morphine concentration, pain level, combination of local anesthetics, and all variables that were statistically significant with pruritus were entered into the second step of multivariate logistic regression. The third step was to add pain level and a combination of local anesthetics as the interaction term into the logistic regression model. The fourth step was to demonstrate the effects of pain level and combined local anesthetics as moderators on the association of pruritus and morphine based on the final multivariate logistic regression [21]. Sample size calculation and statistical analysis was performed using R version 3.6.3 (R Foundation for Statistical Computing, Vienna, Austria).

### Sensitivity analysis

In order to reduce possible bias affecting the incidence of pruritus due to uneven distribution of features, we performed a sensitivity analysis using propensity-score approach to match patients (PSM). The variables for logistic-regression based propensity score were age, twin pregnancy, gestational diabetes, hypertension, term delivery, assisted reproductive treatment, primipara, cesarean section history and BMI, which were chosen based on univariate analysis. Matching was performed using nearest neighbour matching with a 1:1 ratio. The same previous analysis was administered to the propensity-score matched data.

## Results

### Participant characteristics

**Fig. 1:**
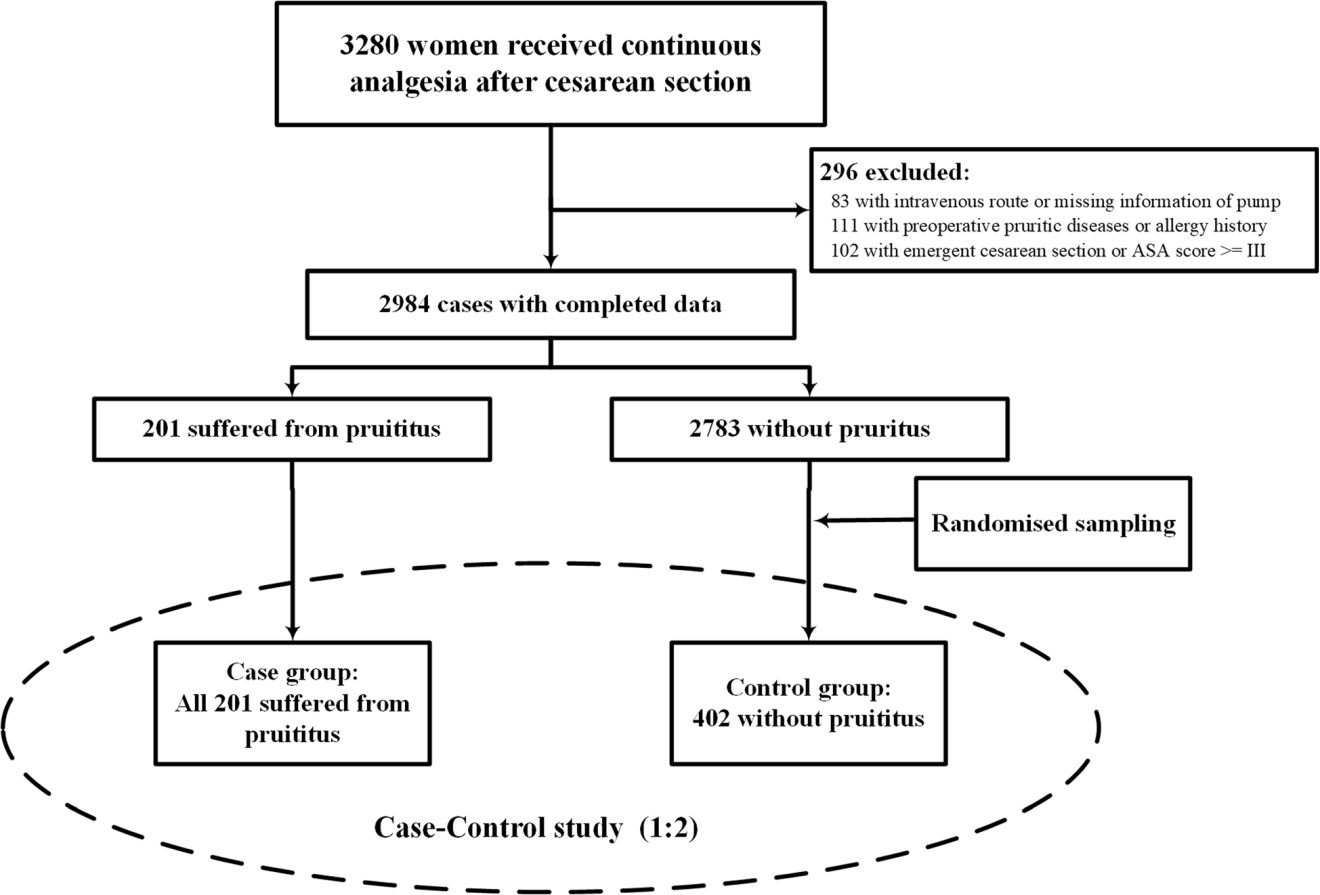
Design and inclusion for the case-control study

Between June 2011 and March 2014, a total of 3280 women used a patient-controlled analgesia pump after cesarean section. Based on the exclusion criteria, 296 women were excluded due to emergent cesarean section, ASA grade III or higher, or incomplete information regarding previous pruritus-related diseases (Fig. 1). As a result, information from 2984 patients was collected, of whom 201 reported pruritus in the hospital and, thus, comprised the case group. A double-size (i.e., *n* = 402) random sample was selected as the control group among women without pruritus using computer-generated random numbers (Fig. 1).

### Univariate analysis

Among the 603 participating women, the range of morphine concentration ranged from 10 to 40 μg/mL (median 20.00 μg/mL [IQR 20–30 μg/mL]). In the case group, the median morphine concentration was 20 μg/mL (IQR 20.00–30.00 μg/mL), while it was 25 μg/mL (IQR 20.00–30.00 μg/mL) in the control group; the difference was statistically significant.

In the pruritus group, 89.9% received morphine combined with 0.15% ropivacaine analgesia, and 10.1% received morphine combined with 0.15% levobupivacaine.

The median pain level was mild (range from none to severe). There was no significant difference in the severity of pain between the pruritus and control groups.

#### Other variables

Nearly one-half of the women were primiparas (43.8% [pruritus group], 58.0% [control group]), and 5.1% underwent assisted reproductive treatment for the index pregnancy, with significant differences between the pruritus group and the control group (8.5% vs. 3.5%, respectively). One-third of the women had a history of cesarean section (37.3% [pruritus group] vs. 28.4% [control group]). The most common ASA scores was 1. Other variables were not statistically significant between the groups (Table 1).

**Table 1:** Characteristics of participants

		Full Sample (***N***=603) n(%)	Pruritus (***N***=201) n(%)	Control (***N***=402) n(%)	p
Age (mean (SD))		29.08 (4.26)	29.71 (4.03)	28.77 (4.34)	0.011
Higher age	< 35y	531 (88.1)	172 (85.6)	359 (89.3)	0.231
	>= 35y	72 (11.9)	29 (14.4)	43 (10.7)	
Local anesthetics	ropivacaine	541 (89.9)	176 (88.0)	365 (90.8)	0.354
	levobupivacaine	61 (10.1)	24 (12.0)	37 (9.2)	
Morphine concentration (median [IQR])		20.00 [20.00, 30.00]	25.00 [20.00, 30.00]	20.00 [20.00, 25.00]	< 0.001
ASA score	I	390 (64.7)	134 (66.7)	256 (63.7)	0.527
	II	213 (35.3)	67 (33.3)	146 (36.3)	
Twin pregnancy	single	561 (93.0)	184 (91.5)	377 (93.8)	0.396
	twin	42 (7.0)	17 (8.5)	25 (6.2)	
Gestational diabetes	No	525 (87.1)	177 (88.1)	348 (86.6)	0.699
	Yes	78 (12.9)	24 (11.9)	54 (13.4)	
Hypertension	No	568 (94.2)	191 (95.0)	377 (93.8)	0.666
	Yes	35 (5.8)	10 (5.0)	25 (6.2)	
Term delivery	No	98 (16.3)	36 (17.9)	62 (15.4)	0.507
	Yes	505 (83.7)	165 (82.1)	340 (84.6)	
Assisted reproductive treatment	No	572 (94.9)	184 (91.5)	388 (96.5)	0.016
	Yes	31 (5.1)	17 (8.5)	14 (3.5)	
Primipara	No	282 (46.8)	113 (56.2)	169 (42.0)	0.001
	Yes	321 (53.2)	88 (43.8)	233 (58.0)	
Cesarean section history	No	414 (68.7)	126 (62.7)	288 (71.6)	0.032
	Yes	189 (31.3)	75 (37.3)	114 (28.4)	
BMI (median [IQR])		26.70 [24.70, 28.90]	26.50 [24.60, 28.80]	26.80 [24.70, 29.10]	0.402
Pain level	None	149 (24.8)	49 (24.4)	100 (24.9)	0.978
	Mild	413 (68.6)	139 (69.2)	274 (68.3)	
	Moderate	40 (6.6)	13 (6.5)	27 (6.7)	

### Multivariate logistic regression

Univariate analysis suggested that higher morphine concentration, assisted reproductive treatment, and primipara and cesarean section history were significantly more prevalent in the pruritus group than in the control group. Due to a high correlation coefficient between primipara and cesarean section history (*r* = 0.72, *P* < 0.01), cesarean section history was dropped in the multivariate logistic model. The final logistic model revealed that, with the interaction terms of morphine concentration, pain level, and local analgesics added, some of the original associations had changed (Table 2).

**Table 2:** Logistic models for pruritus

Models	Independents (reference)	OR	95% CI
**Model 1(series)**			
Model 1a	Higher age (<35y)	1.408	(0.697~2.118)
Model 1b	Local anesthetics (ropivacaine)	1.345	(0.613~2.077)
Model 1c	Morphine concentration (continuous)	1.084***	(1.046~1.121)
Model 1d	ASA score (1)	0.877	(0.564~1.190)
Model 1e	Twin pregnancy (single)	1.393	(0.500~2.286)
Model 1f	Gestational diabetes (No)	0.874	(0.425~1.323)
Model 1 g	Hypertension (No)	0.79	(0.194~1.385)
Model 1 h	Term delivery (No)	0.836	(0.459~1.213)
Model 1i	Assisted reproductive treatment (No)	2.561**	(1.694~4.427)
Model 1j	Primipara (No)	0.565***	(0.372~0.758)
Model 1 k	Cesarean section history (No)	1.504**	(1.064~2.043)
Model 1 l	BMI (continuous)	0.962	(0.912~1.013)
Model 1 m	Pain level mild (none)	1.035	(0.623~1.447)
Model 1n	Pain level moderate (none)	0.983	(0.251~1.714)
**Model 2**			
	Morphine concentration	1.166***	(1.121~1.212)
	Pain level mild (none)	1.041	(0.583~1.498)
	Pain level moderate (none)	0.642	(0.087~1.197)
	Local anesthetics (ropivacaine)	1.058	(0.388~1.727)
	Assisted reproductive treatment (No)	5.897***	(1.086~10.709)
	Primipara (No)	0.427***	(0.260~0.594)
**Model 3**			
	Morphine concentration	1.014	(0.834~1.194)
	Pain level mild (none)	22.153***	(15.132~29.173)
	Pain level moderate (none)	3.047	(0.932~10.025)
	Local anesthetics (ropivacaine)	5.227	(0.174~27.627)
	Assisted reproductive treatment (No)	5.137***	(2.159~8.115)
	Primipara (No)	0.424***	(0.256~0.592)
	Morphine concentration: ropivacaine: pain level none	1.214*	(1.071~1.456)
	Morphine concentration: levobupivacaine: pain level none	1.072	(0.867~1.276)
	Morphine concentration: ropivacaine: pain level mild	1.163	(0.940~1.387)
	Morphine concentration: levobupivacaine: pain level mild	1.014	(0.843~1.185)
	Morphine concentration: ropivacaine: pain level moderate	1.114*	(1.018~1.251)
	Morphine concentration: levobupivacaine: pain level moderate	NA	NA

Model 1 (series): univariate logistic regression

Model 2: multivariate logistic regression (no interaction terms)

Model 3: multivariate logistic regression with interaction between morphine, local anesthetics and pain level

*p < 0.05

** *p*< 0.01

*** *p*< 0.001

**Fig. 2:**
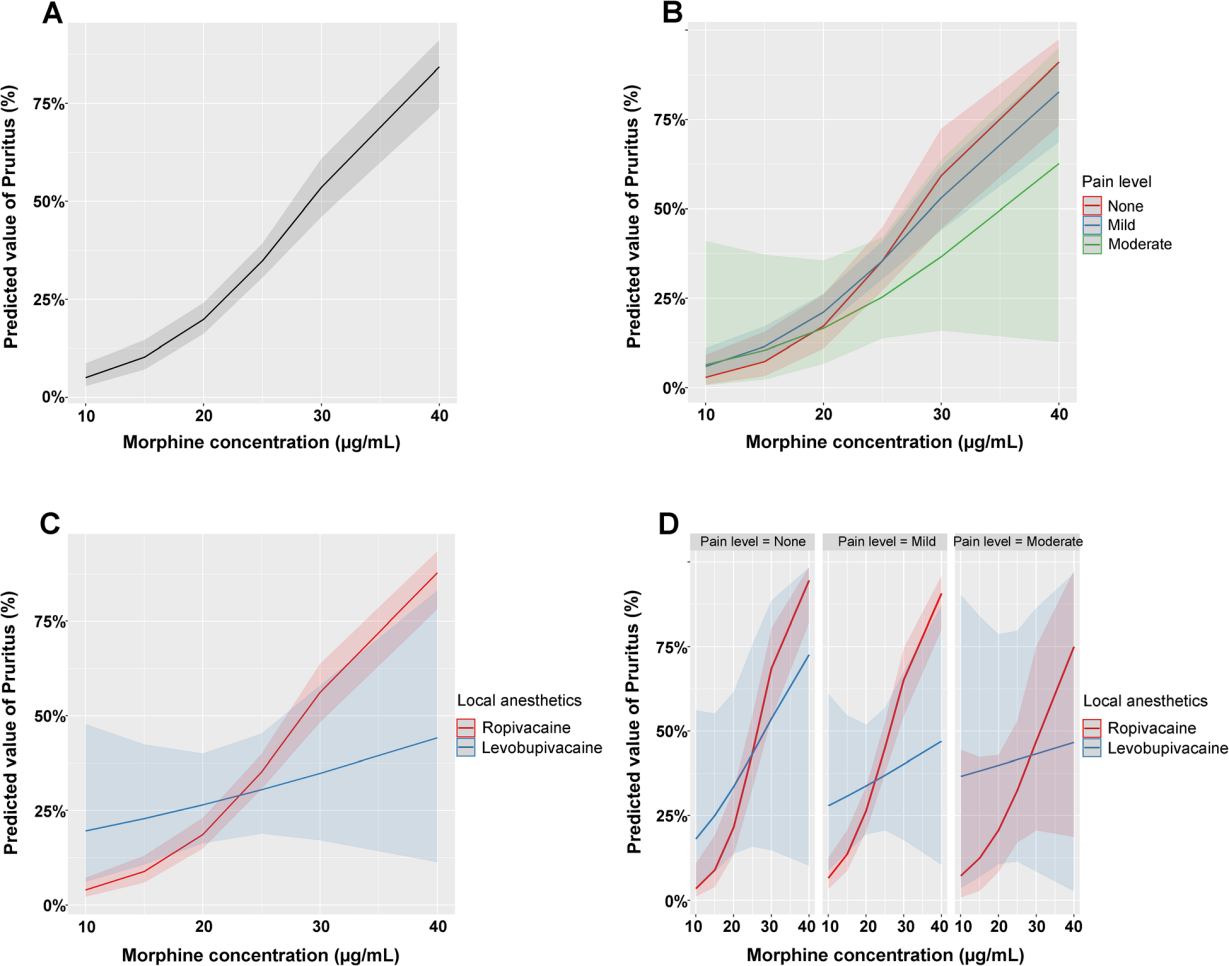
**a** Overall association between morphine concentration and pruritus. **b** Moderation effects of pain level on the association between morphine concentration and pruritus. **c** Moderation effects of local anesthetics on the association between morphine concentration and pruritus. **d** Moderation effects of combined local anesthetics and pain level on the associations between morphine concentration and pruritus (based on Model 3)

The effects of pain level and the combination of local anesthetics as moderators on the associations between pruritus and morphine based on the final model 3 are shown in Fig. 2. The higher the morphine level, the higher the incidence of pruritus (Fig. 2a); however, in each stratum of pain level or local anesthetics in combination, the effect was different.

In general, the higher the morphine concentration, the higher the probability of pruritus occurrence (Fig. 2a). With morphine concentrations from 10, 15, 20, 25, 30 to 40 μg/mL, the probability of pruritus increased sequentially from 0.05, 0.1, 0.2, 0.35, 0.54 to 0.84, respectively. Within the stratum with pain level = none, the tendency was steepest compared with other strata (Fig. 2b). Within the stratum of the local anesthetic levobupivacaine, the tendency was flatter compared with that of ropivacaine (Fig. 2c).

Considering both pain level and local anesthetics as moderators (Fig. 2d), within the stratum of pain level = none, the steep tendency of increased occurrence of pruritus with elevated morphine concentration was similar between the ropivacaine and levobupivacaine strata. The incidence of pruritus increased from 0.03 (95% CI of 0.01–0.11) in the morphine 10 μg/mL group to 0.96 (95% CI of 0.86–0.99) in the morphine 40 μg/mL group when combined with ropivacaine, while the incidence of pruritus increased from 0.19 (95% CI of 0.04–0.59) in 10 μg/mL morphine group to 0.83 (95% CI of 0.17–0.99) in 40 μg/mL morphine group when combined with levobupivacaine.

With pain levels mild or moderate, the trend remained steep in the ropivacaine stratum, while it became significantly flatter when combined with levobupivacaine. In the stratum of pain level = mild combined with levobupivacaine, the incidence of pruritus increased from 0.33 (95% CI 0.1–0.68) in the 10 μg/mL morphine group to only 0.48 (95% CI 0.1–0.88) in the 40 μg/mL morphine group. In the stratum of the pain level = moderate combined with levobupivacaine, the incidence increased from 0.4 (95% CI 0.04–0.92) to only 0.56 (95% CI 0.03–0.98).

### Sensitivity analysis

Sensitivity analysis with propensity-score matched participants yielded a 1:1 sample of 181 in the case group and 181 in control group. The results of the multivariate analysis in the propensity-score matched participants were in consistent with above findings.

The higher the morphine level, the higher the incidence of pruritus, and the effect was different in each stratum of pain level or local anesthetics in combination. Within the stratum with pain level = none, the tendency was steepest compared with other strata. Within the stratum of the local anesthetic levobupivacaine, the tendency was flatter compared with that of ropivacaine. The incidence of pruritus increased from 0.05 (95% CI of 0.01–0.19) in the morphine 10 μg/mL group to 0.97 (95% CI of 0.84–0.99) in the morphine 40 μg/mL group when combined with ropivacaine, while the incidence of pruritus increased from 0.27 (95% CI of 0.04–0.74) in 10 μg/mL morphine group to 0.82 (95% CI of 0.08–1.00) in 40 μg/mL morphine group when combined with levobupivacaine. With pain levels mild or moderate, the trend became significantly flatter, especially when combined with levobupivacaine.

## Discussion

The association between the occurrence of pruritus and morphine consumption in epidural morphine analgesia after cesarean section is a major clinical problem [19]. The primary finding of our case-control study was the direct association between morphine concentration and risk for pruritus. The risk for pruritus increased significantly as morphine concentration increased within the 10 to 40 μg/mL interval. Although epidural morphine is widely used for analgesia after cesarean section, few relevant investigations have defined pruritus as the primary outcome to study its association with epidural administration of morphine. In 1988, Fuller [22] reviewed 4880 women undergoing cesarean section with an epidural morphine dose of 2, 2.5, 3, 3.5, 4, 4.5, and 5.0 mg, and suggested that increasing the dose of epidural morphine could increase the incidence of pruritus. In 2013, Singh [23] randomly assigned 90 women to receive 3 mg or 1.5 mg epidural morphine, and found that the incidence of severe pruritus was lower in the 1.5 mg epidural morphine group (relative risk 0.44 vs. 0.41), providing non-inferior analgesia. In these studies, pruritus was only an indicator of side effects instead of the primary outcome; therefore, the power of the studies to identify the association between pruritus and morphine was insufficient.

Previous studies have suggested possible complex relationships between the use of analgesics, and pain-relieving effects, and side effects [10, 24]. Pruritus and pain may be conducted through similar anatomical and functional neuronal pathways and, therefore, interact closely. The most influential explanation for the relationship between pain and pruritus is that pruritus receptors are part of pain receptors, and activation of pain signals help to inhibit pruritus signal circuits [25]. Consequently, some studies have suggested that opioid-related pruritus may be due to the indirect effects of analgesic efficacy, and inhibition of pain may evoke pruritus [26]. The interaction between attenuated pain and increased pruritus behavior(s) has been confirmed in recent studies [27]. Another key question in our study was to explore whether pain level and combination medication can affect the association between morphine concentration and the incidence of pruritus. It was found that the association between morphine concentration and risk for pruritus was most likely moderated by a joint, complex combination of other clinical features. When the pain level was high, the trend in the incidence of pruritus increased with morphine concentration flattened significantly, compared with low pain level.

Furthermore, this study demonstrated that morphine combined with ropivacaine or levobupivacaine could moderate the effect on the association between morphine dosage and pruritus. Compared with ropivacaine, the association between increase in pruritus and morphine concentration flattened significantly when combined with levobupivacaine. Currently, ropivacaine and levobupivacaine are the most commonly used analgesics. Ropivacaine is believed to exert selective effects on sensory fibers, has a stronger ability to inhibit nerve conduction in pain-sensitive fibers, and exhibits less mobility reduction [28, 29]. Levobupivacaine is a bupivacaine S(−) isomer that has an onset and duration of action similar to bupivacaine, but with fewer toxic effects on the cardiovascular and central nervous systems [30, 31]. Although levobupivacaine may have a slower onset, it exerts higher motor block than ropivacaine [29]. The combination of local anesthetics may influence the pain-relieving effect or morphine and, thus, indirectly moderated the occurrence of pruritus. However, due to the small proportion of levobupivacaine used in the study sample and a small proportion of higher pain levels (i.e., pain level ≥ moderate), a very small sample size in the strata or cells in the contingency table may have led to insufficient statistical power for post hoc analysis, which in turn may have resulted in wider confidence intervals in the moderation analysis.

Our study observed that undergoing IVF treatment might be a risk factor for the pruritus. However, at present, there is no scientific evidence to explicitly explain the mechanism between IVF and pruritus after epidural morphine for post-cesarean analgesia. Sheiner et al. [32] found that fertility treatment was a risk factor for pruritus gravidarum. Bolukbas [33] explored that higher rates of hormone treatment during IVF pregnancy may increase the incidence of intrahepatic cholestasis of pregnancy (ICP) characterized by elevation of serum bile acid and skin pruritus. Arrese M [34] suggested that estrogen could cause ICP by modifying the fluidity of plasma membrane. In our study, we excluded cases with preoperative pruritic diseases such as ICP and observed that IVF was a potential risk factor of pruritus after epidural morphine. More researches are needed to investigate, especially the role of hormone treatment plays in this association.

In this study, the incidence of pruritus was lower in primiparas. However, findings reported in the literature are not consistent. Recently, Tan [35] reported that primipara or multipara were not associated with pruritus caused by epidural injection of morphine for analgesia after cesarean section. Sheiner et al. [32] compared obstetric risk factors and pregnancy outcomes in women with pruritus and found that primipara women had a higher risk for experiencing pruritus during pregnancy (odds ratio 1.3 [95% CI 1.1–1.7]; *p* = 0.014). Existing research suggests that estrogen content gradually increased from early pregnancy to late pregnancy [36], and pruritus after neuraxial opioid may be related to the interaction between opioid receptors and increased estrogen levels [37]. We speculate that there were some discrepancies between primipara and multipara in pruritus sensitivity and hormone levels. Nevertheless, prospective cohort studies are needed to examine the relationship between parity and pruritus.

The present study had some limitations, the first of which was its single-center, retrospective design, which limits the generalizability of the results and its ability to draw causal inferences. Second, pruritus is a subjective sensation; thus, it is difficult to avoid response bias caused by self-reporting. Third, some information, such as site of pruritus, and/or duration and grade or treatment of pruritus, may have been incomplete in our electronic record system. However, this should not have affected the results because the purpose of the analysis was to examine the associations between pruritus and morphine rather than to provide an estimate of pruritus rate. Although the sample size in this study was larger than those of similar studies, the sample, when divided into subgroups of various combinations of interactive factors, may not have been sufficient, which limited the statistical power of further analysis to explore potential confounding or moderation. Notwithstanding these limitations, our study contributes to the scarce literature exploring the association between pruritus and morphine along with other risk factors.

## Conclusions

The results of our study demonstrated that higher concentrations of morphine, multipara, and assisted reproductive treatment were associated with a higher probability of pruritus. The associations persisted, even after the effects of pain level and local anesthetics used in combination were taken into consideration. Future studies should aim to identify and examine more potential risk factors and adopt a prospective design to explore and verify a wider mixture of factors. The current practice of obstetrical analgesia is to encourage the use of local anesthetics in combination with adjuvant drugs to maintain efficacy and comfort while reducing the dosage of individual drugs to avoid side effects. Results of the present study may guide clinical practitioners in selecting appropriate opioid concentrations and optimal drug combinations for obstetrical analgesia.

## Data Availability

The data used and/or analyzed during the current study are available from the corresponding author on reasonable request.

## Abbreviations

ASA: American Society of Anesthesiologist
CI: confidence interval
IQR: interquartile range
IVF: in vitro fertilization
SD: standard deviation
